# Clinical effects of laparotomy with perioperative continuous peritoneal lavage and postoperative hemofiltration in patients with severe acute pancreatitis

**DOI:** 10.1186/1749-7922-4-45

**Published:** 2009-12-16

**Authors:** Roberto Caronna, Michele Benedetti, Andrea Morelli, Monica Rocco, Loretta Diana, Giampaolo Prezioso, Maurizio Cardi, Monica Schiratti, Gabriele Martino, Gianfranco Fanello, Federica Papini, Francesco Farelli, Roberto L Meniconi, Michele Marengo, Giuseppe Dinatale, Piero Chirletti

**Affiliations:** 1 University of Rome "La Sapienza", Department of Surgery "Francesco Durante", General Surgery N, Viale del Policlinico 155, Rome, 00161, Italy; 2 Residency Program in General Surgery V, University of Rome "La Sapienza", Viale del Policlinico 155, Rome, 00161, Italy; 3 Istituto Superiore di Sanità, Viale Regina Elena 299, Rome, 00161 Italy; 4 University of Rome "La Sapienza", Intensive Care Unit, Viale del Policlinico 155, Rome, 00161, Italy

## Abstract

**Background:**

The elevated serum and peritoneal cytokine concentrations responsible for the systemic response syndrome (SIRS) and multiorgan failure in patients with severe acute pancreatitis lead to high morbidity and mortality rates. Prompted by reports underlining the importance of reducing circulating inflammatory mediators in severe acute pancreatitis, we designed this study to evaluate the efficiency of laparotomy followed by continuous perioperative peritoneal lavage combined with postoperative continuous venovenous diahemofiltration (CVVDH) in managing critically ill patients refractory to intensive care therapy. As the major clinical outcome variables we measured morbidity, mortality and changes in the Acute Physiology and Chronic Health Evaluation (APACHE II) score and cytokine concentrations in serum and peritoneal lavage fluid over time.

**Methods:**

From a consecutive group of 23 patients hospitalized for acute pancreatitis, we studied 6 patients all with Apache II scores ≥19, who underwent emergency surgery for acute complications (5 for an abdominal compartment syndrome and 1 for septic shock) followed by continuous perioperative peritoneal lavage and postoperative CVVDH. CVVDH was started within 12 hours after surgery and maintained for at least 72 hours, until the multiorgan dysfunction syndrome improved. Samples were collected from serum, peritoneal lavage fluid and CVVDH dialysate for cytokine assay. Apache II scores were measured daily and their association with cytokine levels was assessed.

**Results:**

All six patients tolerated CVVDH well, and the procedure lasted a mean 6 days (range, 3-12). Five patients survived and one died of Acinetobacter infection after surgery (mortality rate 16.6%). The mean APACHE II score was ≥ 19 (range 19-22) before laparotomy and decreased significantly during peritoneal lavage and postoperative CVVDH (P = 0.013 by matched-pairs Students *t*-test). The decrease in cytokine concentrations in serum and lavage fluid was associated with the decrease in APACHE II scores and high interleukin 6 (IL-6) and tumor necrosis factor (TNF) concentrations in the hemofiltrate.

**Conclusion:**

In critically ill patients with abdominal compartment syndrome, septic shock or high APACHE II scores related to severe acute pancreatitis, combining emergency laparotomy with continuous perioperative peritoneal lavage followed by postoperative CVVHD effectively reduces the local and systemic cytokines responsible for multiorgan dysfunction syndrome thus improving patients' outcome.

## Background

In patients with severe acute pancreatitis (SAP), the primary cause of morbidity, mortality and high costs is the multiorgan dysfunction syndrome (MODS) [1,2]. Accumulating evidence underlines the relationship between sepsis, systemic multiorgan damage (lung, liver, kidney, and heart) and elevated serum and peritoneal concentrations of cytokines (IL-1, IL-6, IL-8, IL-10) and tumor necrosis factor (TNF) [3-12]. A procedure known to reduce plasma cytokine levels is continuous venovenous diahemofiltration (CVVDH) [13,14]. As well as purifying the blood, hemofiltration has a major adjunctive therapeutic role as immunomodulatory therapy in sepsis [15,16]. The high levels of inflammatory mediators (cytokines and others) found not only in serum but also in peritoneal fluid from patients with SAP underline the importance of reducing cytokine levels in the SAP-related systemic inflammatory response syndrome (SIRS) [2,17,18].

In 20-30% of patients manifestingprogressive multiorgan failure, intensive care treatment fails and mortality reaches 40% [19]. In these critically ill patients, severe complications such as abdominal compartment syndrome or sepsis often necessitate emergency laparotomy [20,21]. Prompted by reports underlining the importance of reducing circulating inflammatory mediators in severe acute pancreatitis [3,22-28], we conjectured that peritoneal and systemic cytokine concentrations could be reduced by combining emergency laparotomy with continuous perioperative peritoneal lavage with postoperative CVVDH. Lowering local and systemic cytokine toxicity might thus reduce morbidity and mortality in acute pancreatitis.

Our aim in this preliminary single-center study was to find out whether in a small series of selected critically ill patients with SAP refractory to ICU therapy a new approach comprising emergency laparotomy to resolve abdominal compartment syndrome or sepsis followed by continuous perioperative peritoneal lavage to remove local cytokines and postoperative CVVDH to reduce systemic cytokines would benefit patients' outcome. As outcome variables we evaluated postoperative IL-6 and TNF concentrations in serum, peritoneal lavage outflow and CVVDH filtrate and sought an association between their decrease and changes in the clinical progression of SAP over time as measured by APACHE II scores.

## Methods

We studied 23 consecutive patients with acute pancreatitis diagnosed according to the Italian Association for the Study of the Pancreas (AISP) criteria [29]. The severity of acute pancreatitis was classified according to the Atlanta criteria [30]. The major cause of acute pancreatitis was biliary disease (20 patients) followed by alcohol (2 patients) and hyperlipidemia (1 patient). Of the 23 patients enrolled, 18 had mild acute pancreatitis but 5 had severe acute pancreatitis on presentation. According to the Balthazar computed tomographic (CT) criteria for grading acute pancreatitis [31] 12 patients were in grade C, 8 in grade D and 3 in grade E (severe pancreatitis).

At admission all patients underwent standard medical therapy according to the guidelines of the Italian Association for the Study of the Pancreas (AISP) [32] including fluid resuscitation, gastrointestinal decompression, oxygen therapy, parenteral nutritional support, somatostatin, antibiotics (imipenem-cilastatin) and infusion of the enzyme inhibitor gabexate mesilate (900 mg/day). Of the 23 initial participants, 17 patients responded well to medical therapy and were discharged after a mean 13 days. The remaining 6 patients (2 men and 4 women; mean age 60.8 years, range 27-74) whose clinical conditions failed to improve or worsened after therapy lasting 48 hours all had an Apache II score of ≥ 19. These 6 patients underwent emergency laparotomy, 5 for an abdominal compartment syndrome, defined as a susteined intraabdominal pressure about 20 mmHg associated with new organ failure, and 1 for septic shock. At surgery the anterior pancreatic wall was widely exposed, the capsule fully opened and Kocher's maneuver was used to mobilize the pancreatic head and body anteriorly. The pancreatic body and tail were then manually freed starting from the Treitz ligament. Eventual necrotic tissue and fluid collections were sampled for microbiological cultures and removed. Patients with acute biliary pancreatitis underwent cholecystectomy and a biliary drain was placed through the cystic duct. To allow complete lavage, drains were placed close to the anterior and posterior pancreatic walls, in the paracolic gutters and pelvis. A lavage solution containing 6 to 8 liters of normal saline and gabexate mesilate (1000 mg) was perfused through the drains every 24 hours for at least 7 days.

After surgery all six patients were admitted to the ICU and CVVDH was started within 12 hours. For vascular access, a double coaxial lumen 14-Fr catheter was inserted percutaneously through the right internal jugular or femoral vein using the Seldinger technique. A Baxter BM25 system (Baxter, USA) was used for CVVDH with a polyacrylonitrile NA69 hemofilter (1.2 m^2^surface area, 35-kD limit; Hospal, USA). Blood flow was set at 50-75 ml/min and ultrafiltrate flow at 1000 ml/h, transmembrane pressure was maintained between 450-460 mmHg, and the replacement fluid was pre-diluted and infused. Low-molecular-weight heparin was used as the anticoagulant, patient-activated clotting time was adjusted to 60-70 seconds, and a strictly neutral balance was maintained using a digital balance system (Baxter). CVVDH was maintained for a mean 6 days (range 3-8). The AN69 hemofilter (1.2 m^2^) was changed every 24 hours.

Samples for measuring cytokine concentrations were collected from serum at admission (T0) and 48 hours later (T48). After surgery, samples were taken also from peritoneal lavage fluid and hemofiltrate on postoperative days I, IV, VII, and XIV. The last sample was collected when CVVDH ended. IL-6 and TNF were assayed with an enzyme-linked immunosorbent assay (ELISA) kit using the quantitative immunoenzymatic sandwich method.

Vital signs, including body temperature, breath rate, blood pressure and heart rate were recorded half hourly. Blood samples were collected every 24 hours to monitor blood cell counts, serum amylase and electrolytes, liver and kidney function, and arterial blood gases. As an outcome variable, APACHE II scores were determined daily to evaluate the patients' clinical conditions and were matched with IL-6 and TNF concentrations in serum and lavage fluid. The other outcome variables assessed were surgical morbidity and mortality.

### Statistical analysis

Results are expressed as mean ± SD and data for the two continuous outcome variables were analyzed using Student's matched-pairs *t*-test. Differences were considered significant at *P *< 0.05.

## Results

Of the six patients with severe acute pancreatitis who had emergency laparotomy followed by continuous perioperative peritoneal lavage with postoperative CVVDH, five were cured and discharged from hospital. One patient died of septic shock related to Acinetobacter infection (surgical mortality 16.6%). None of the patients had major surgery-related complications during the postoperative course except an enteric fistula that developed in 1 patient and responded to conservative therapy with prolonged total parenteral nutrition. The mean hospital stay was 28.5 days, and 13.3 days in the ICU. Cultures of microbiological samples taken during surgery grew Enterococcus in 2 cases, Escherichia coli in 2, Pseudomonas in 1 and no infection in another.

**Figure 1:**
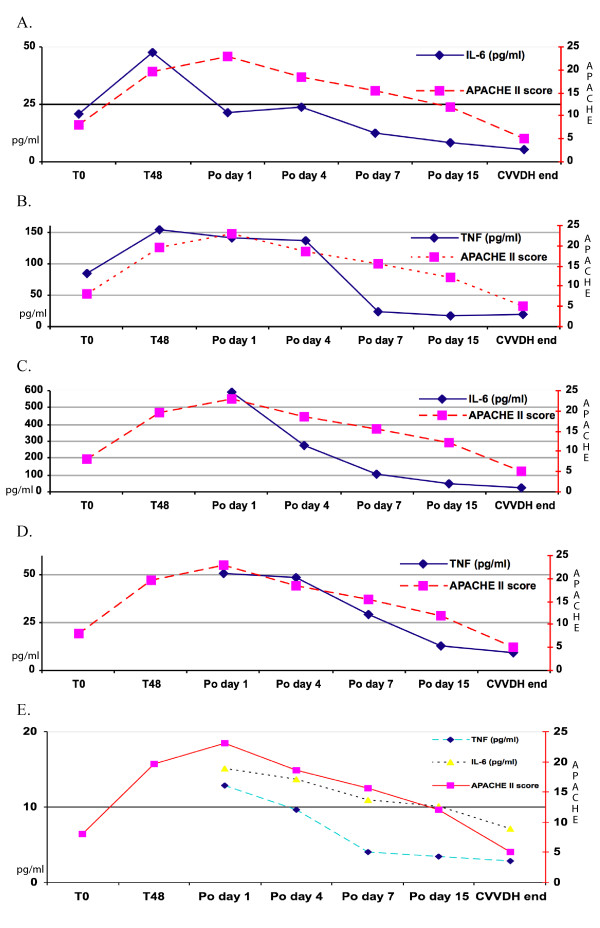
**Panel A and B**. Note the high IL-6 and TNF serum concentrations before surgery (T0 and T48) and the rapid decrease during peritoneal lavage and continuous venovenous diahemofiltration (CVVDH). APACHE II scores improved significantly from T48 to the end of CVVDH (p = 0.013 by matched-paired Student's t-test) whereas IL-6 and TNF concentrations decreased over the same time course though not significantly. Panel C and D. The decrease in IL-6 and TNF concentration in peritoneal lavage fluid became significant (P = 0.019 and P = 0.008) between the two time-points T48 and when CVVDH ended and was significantly associated with the decrease in APACHE II scores over the same time course (P = 0.013). Panel E. Note the high IL-6 and TNF concentrations in the hemofiltrate, suggesting that continuous venovenous diahemofiltration (CVVDH) effectively purified these patients' sera.

IL-6 and TNF concentrations were high in serum before surgery (T0, Figure 1, panel A and B) and in peritoneal fluid on postoperative day I (Figure 1, panel C and D) but decreased rapidly during peritoneal lavage (Figure 1, panels A, B, C and D). Over the same time course, IL-6 and TNF concentrations in the hemofiltrate increased (Figure 1, panel E).

The other outcome variable, the mean Apache II score measuring patients' worsening clinical conditions, increased from 8 at admission to 19.6 at 48 hours to 23 on postoperative day I when CVVDH began. Conversely, it decreased significantly during peritoneal lavage and CVVDH (from 18.6 on postoperative day IV to 8 after the second postoperative week) (P = 0.013) as patients' clinical conditions improved over time. Over the same time course, IL-6 and TNF concentrations in serum remained statistically unchanged (P = 0.07 and P = 0.309) but in peritoneal lavage fluid decreased significantly (P = 0.019 and 0.008), whereas hemofiltrate TNF concentrations alone increased (P = 0.03) (Figure 1, panel E). Student's t-test for matched pairs disclosed a significant association between decreasing cytokine concentrations in serum and peritoneal lavage fluid and decreasing Apache II scores (Figure 1, panel A-E).

The only CVVDH-related adverse reaction was a high fever caused by an infected catheter that resolved when the catheter was removed. Hypophosphatemia developed in two patients and was normalized by increasing the phosphate content in the CVVDH dialitic solution.

## Discussion

Our promising results in this single-center preliminary study in selected severely ill patients refractory to ICU therapy suggest that the new approach we propose, emergency laparotomy to resolve abdominal compartment syndrome or sepsis followed by continuous perioperative peritoneal lavage and postoperative CVVDH, successfully reduces local and systemic cytokines thus reducing morbidity and mortality and improving patients' clinical outcome without increasing the high surgery-related morbidity.

All six patients with SAP in this series had high APACHE II scores before surgery, indicating MODS [33] and they also had severe SAP-related complications, in five patients an abdominal compartment syndrome and in one sepsis, all refractory to ICU therapy and therefore necessitating emergency surgery. In patients with SAP, the inflammatory response (SIRS) to extensive peripancreatic and retroperitoneal fatty tissue damage, may lead to sepsis, acute respiratory distress syndrome (ARDS), acute renal failure, hypovolemic shock, acute liver failure and MODS, now the primary cause of morbidity and mortality in SAP [13].

In accordance with their severe clinical presentation, MODS and multiorgan failure, all our patients with SAP had high perioperative IL-6 and TNF concentrations in serum, peritoneal lavage outflow and CVVDH hemofiltrate, presumably related to pro-inflammatory cytokine release and neutrophil activation [22,23]. Although we measured only IL-6 and TNF, other inflammatory mediators known to play a critical role in acute pancreatitis and MODS include TNFa, IL-1b, IL-6, IL-8, platelet-activating factor, and IL-10 [3,22-28].

Our study extends to the few therapeutic options for removing the inflammatory mediators responsible for SAP-related systemic complications [33]. In all 6 patients we treated, anti-inflammatory therapy using continuous perioperative peritoneal lavage and postoperative CVVDH effectively reduced IL-6 and TNF concentrations in peritoneal lavage fluid and serum. Continuous hemofiltration (CHF), especially CVVDH, was developed for patients in severe conditions and has been used mainly in critical care [34,35]. CVVDH can remove many inflammatory mediators, includingTNF, IL-1, IL-6, sIL-2R, IL-8, IL-2 and IL-10 all having a molecular weight lower than 50000 Daltons [1,36-40]. CVVDH also helped to normalize our patients' water, electrolyte and acid-base balance and homeostasis related to renal dysfunction.

In line with others, we provide further evidence that continuous perioperative peritoneal lavage reduces cytokine concentrations in the abdominal cavity and diminishes their systemic absorption thus halting the progression of SIRS and MODS [2,18]. The higher the cytokine concentrations in the peritoneal cavity the greater is the quantity absorbed into the blood. In an experimental model of acute pancreatitis Mikami et al found increased IL-1β and TNF-α levels in the lavage fluids in all models during the first 6 hours after induction, and the peak levels accorded with the severity of pancreatitis [18]. In a large study, including 577 patients, Dugerneir et al observed significantly lower mortality for acute pancreatitis in patients who underwent surgical treatment with postoperative peritoneal lavage than in others who had surgery alone (mortality 24.3% vs 43.2%) [2]. An early study already showed that peritoneal lavage had a role in the treatment of acute pancreatitis even before "cytokine storm" became a recognized feature in the pathogenesis of acute pancreatitis [41].

By diluting local peritoneal cytokine concentrations as well as reducing serum reabsorbtion, peritoneal lavage during laparotomy with or without necrosectomy followed by CVDDH presumably had a dual advantage, interfering at two distinct levels in the cytokine-related pathophysiological mechanisms in patients with SAP. When we investigated the association between IL-6 and TNF values in peritoneal lavage fluid and serum and changes in the clinical progression of SAP over time as measured by APACHE II scores, we found elevated APACHE II scores (more than 19) in patients whose serum and peritoneal fluid contained high concentrations of IL-6 and TNF. Conversely, as serum and peritoneal IL-6 and TNF levels decreased our patients' clinical conditions progressively improved (Figure 1, panels A and D) The predicted mortality rate in patients with high APACHE II scores was actually considerably higher than the observed rate (42% vs 16.6%).

During laparotomy, to resolve our patients' life-threatening SAP-related complications, we widely opened the retroperitoneal space and mobilized the pancreas thus extending the surface available for peritoneal cytokine lavage. Although this complex procedure led to no immediate or postoperative complications, the abdominal drains might possibly have caused the abdominal Acinetobacter infection in the patients who died. Conversely, the enteric fistula observed in one case, probably depended on difficulty in dissecting adherences related to a previous surgical intervention.

Whether adding gabexate mesilate to the peritoneal lavage fluid helped to reduce inflammatory mediators in the peritoneal cavity remains unclear. We were prompted to try this inexpensive, non-toxic expedient by evidence from our previous experimental and clinical studies [42,43] showing that this enzyme inhibitor acts directly on pancreatic juice by inhibiting amylase and phospholipase A^2 ^activity.

## Conclusion

The new approach we propose for reducing the circulating cytokines responsible for systemic damage in patients with SAP -- emergency laparotomy followed by continuous perioperative peritoneal lavage and postoperative CVVDH -- is challenging for surgeons, patients and caretakers. In specialized centers, it should nevertheless have a useful place in treating selected critically ill patients with SAP refractory to ICU therapy for whom emergency surgery is needed. By eliminating the local peritoneal cytokines responsible for the development of SIRS and at the same time reducing systemic circulating cytokines from the serum, this management option offers a lower mortality rate than expected and an acceptable clinical outcome. This combined management strategy warrants confirmation in randomized control trials.

## Competing interests

The authors declare that they have no competing interests.

## Authors' contributions

CR and CP designed the study. CR, CP, PG, CM, and SM did surgery. DL conducted the immunoassays. GM, BM, MM, PF, MR, FG, GD and FF helped with patients' postoperative care, data collection and statistical analysis. MA and RC were coordinators in the ICU. All the authors read and approved the final manuscript.
